# Early life stress affects limited regional brain activity in depression

**DOI:** 10.1038/srep25338

**Published:** 2016-05-03

**Authors:** Lian Du, Jingjie Wang, Ben Meng, Na Yong, Xiangying Yang, Qingling Huang, Yan Zhang, Lingling Yang, Yuan Qu, Zhu Chen, Yongmei Li, Fajin Lv, Hua Hu

**Affiliations:** 1 Department of Psychiatry, the First Affiliated Hospital of Chongqing Medical University, Chongqing 400016, P.R. China; 2 Department of Radiology, the First Affiliated Hospital of Chongqing Medical University, Chongqing 400016, P.R. China; 3 Nuclear Medicine Department, Chongqing Cancer Hospital, Chongqing 400030, P.R. China; 4 Department of Psychiatry and Mental Health, Affiliated Hospital of North Sichuan Medical College, Nanchong 637000, P.R. China; 5 Sleep Ward, the First Branch of First Affiliated Hospital of Chongqing Medical University, Chongqing 400016, P.R. China; 6 Sleep and Psychosomatic Diseases Ward, the Third military medical university third hospital, Chongqing 400016, P.R. China; 7 Psychosomatic Department, the Ninth People’s Hospital of Chongqing, Chongqing 400700, P.R. China

## Abstract

Early life stress (ELS) can alter brain function and increases the risk of major depressive disorder (MDD) in later life. This study investigated whether ELS contributes to differences in regional brain activity between MDD patients and healthy controls (HC), as measured by amplitude of low-frequency fluctuation (ALFF)/fractional (f)ALFF. Eighteen first-episode, treatment-naïve MDD patients and HC were assessed with the Childhood Trauma Questionnaire and resting-state functional magnetic resonance imaging. We compared ALFF/fALFF between MDD patients and HC, with or without controlling for ELS, and determined whether ELS level was correlated with regional brain activity in each group. After regressing out ELS, we found that ALFF increased in bilateral amygdala and left orbital/cerebellum, while fALFF decreased in left inferior temporal and right middle frontal gyri in MDD patients relative to controls. ELS positively correlated with regional activity in the left cerebellum in MDD and in the right post-central/inferior temporal/superior frontal cingulate, inferior frontal gyrus and bilateral cerebellum in HC. Our findings indicate that there is only very limited region showing correlation between ELS and brain activity in MDD, while diverse areas in HC, suggesting ELS has few impacts on MDD patients.

Early life stress (ELS) refers to an array of adversities occurring before sexual maturation, including physical, sexual, and emotional abuse, physical and emotional neglect, malnourishment, and loss of a parent^1^,^2^. ELS is highly prevalent and its incidence is higher in psychiatric populations^3^. It is also a risk factor for the development of various disorders such as major depressive disorder (MDD)^4^,^5^.

ELS has been suggested to alter brain structure, including the prefrontal cortex, anterior cingulate cortex (ACC), hippocampus, amygdala, corpus callosum, and cerebellum^6^, as well as ACC-amygdala resting-state functional connectivity^7^ and default mode network (DMN) connectivity^8^. Previous task-based functional magnetic resonance imaging (fMRI) and resting-state blood flow studies have detected abnormal activation of the right hemisphere, cerebellum, basal ganglia, and medial temporal lobe in abused subjects^9^,^10^,^11^,^12^. Some studies focused on effects of life stress on brain in MDD, found early or recent stress may contribute to differences in fronto-limbic structures^13^, as well as prefrontal response to stimuli in MDD^14^,^15^. One fMRI study used a method based on whole brain and found that regional homogeneity decreased in the inferior parietal lobule and superior temporal gyrus in ELS subjects^16^, suggesting that ELS not only affects brain connectivity but also regional activity. The amplitude of low-frequency fluctuation (ALFF) is another parameter for analyzing resting-state fMRI data at the voxel level^17^. ALFF encodes physiologically meaningful indicators of blood oxygen level-dependent (BOLD) variation over time or dynamic fluctuations in intrinsic brain activity in the absence of explicit input, based on the fact that there are coherent low-frequency fluctuating BOLD signals in functionally related brain regions^18^,^19^. Fractional (f)ALFF is defined as the ratio of the power spectrum in the low frequency (0.01–0.08 Hz) range to that of the entire frequency range^20^, and may be regarded as a normalized version of ALFF. The ratio of ALFF/fALFF is a reliable and sensitive measure in the study of healthy^21^, epilepsy^22^, post-traumatic stress disorder (PTSD), and MDD^23^.

Previous ELS-related neuroimaging studies have had various limitations. Firstly, most studies divided participants into those with or without ELS, which was determined based on the Childhood Trauma Questionnaire (CTQ); however, this excluded subjects that had experienced mild ELS^8^,^16^. Other studies did not provide a precise definition for ELS or used other questionnaires to establish ELS^24^. Secondly, many studies focused solely on healthy subjects, which did not enable an examination of the relationship between ELS and MDD^12^,^25^. In addition, most of the earlier studies investigating the effects of abuse used ROI approaches, which are biased towards fronto-limbic systems^7^ rather than taking into consideration the whole brain. Lastly, studies comparing ALFF/fALFF in MDD and healthy controls (HC) disregarded the fact that MDD patients typically score higher for ELS, which may contribute to the observed alterations.

ELS can lead to aberrations in regional brain function that can heighten the risk of MDD in later life. However, not all MDD patients have a history of ELS, and some individuals exposed to ELS are resilient and healthy. It is therefore possible that ELS differentially affects MDD and healthy individuals. The present study investigated regional differences in brain function between MDD and HC as measured by ALFF/fALFF after regression of ELS, and examined the association of ELS with regional cerebral function in MDD and HC to determine differences in patterns of ELS-induced activity.

## Results

### Demographic and clinical information

First-episode, treatment-naïve MDD patients (n = 18, mean age ± SD: 39.28 ± 12.89 years, 13 females, all right-handed), and age-matched HC (n = 18, mean age ± SD: 35.33 ± 10.01 years, 8 females, all right-handed) were included in the study. None of the subjects fell asleep during the scan or had head motion >2 mm or rotation >1° during scanning. Two patients did not complete the CTQ survey; therefore, 16 patients were included in the CTQ correlation analysis. There were no differences between MDD and HC groups in terms of age or years of education. MDD patients had higher CTQ scores (including emotional abuse (EA), physical abuse (PA), sexual abuse, emotional neglect (EN), physical neglect (PN) subscales and total scores; P < 0.05) than HC (Table 1).

### Group differences in spontaneous brain activity

**Figure 1:**
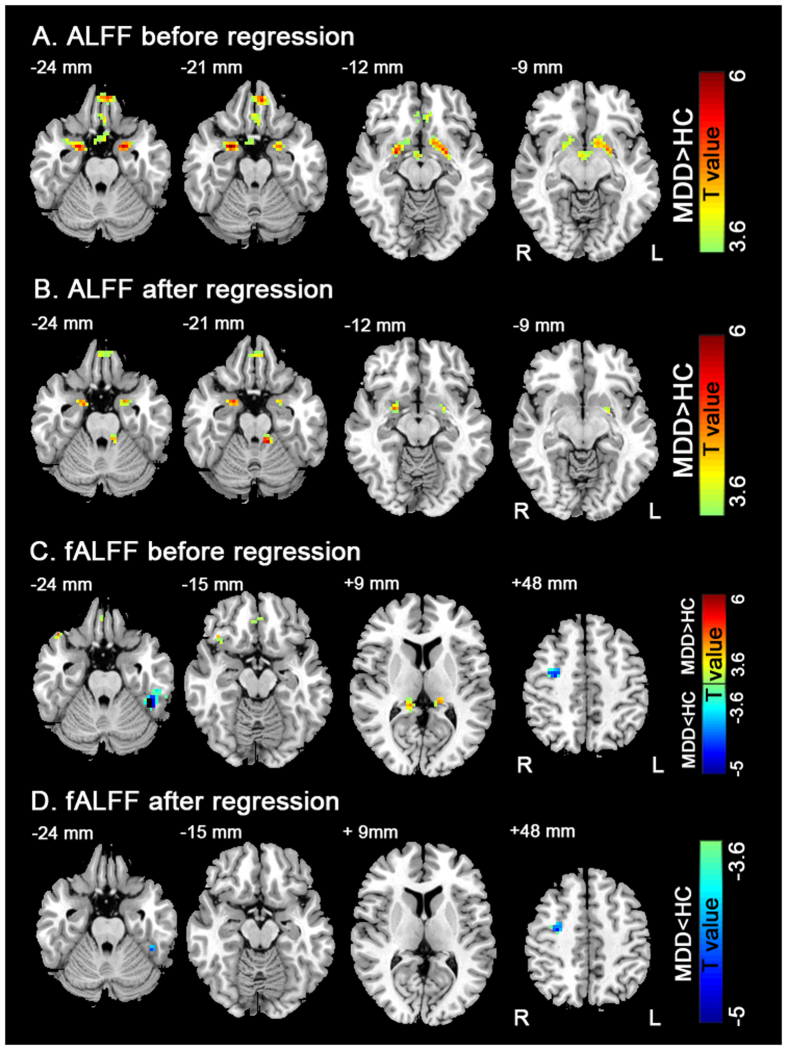
The comparison of regional brain activity t-map between MDD and HC. Figure 1A indicates ALFF differences before CTQ regression. Figure 1B indicates ALFF differences after CTQ regression. Figure 1C indicates fALFF differences before CTQ regression. Figure 1D indicates fALFF differences after CTQ regression. The color coded t-score bars indicate the regional brain activities in these areas of MDD were higher (warm color) and lower (cold color) relative to HC. Left in the figure corresponds to the right side of the brain (*P* < 0.05, AlphaSim corrected).

Compared to the HC group, MDD patients showed higher ALFF in the right and left amygdalae, left orbital gyrus, and left hypothalamus (Fig. 1A and Table 2). After regression of CTQ, MDD patients showed increased ALFF in the right and left amygdalae, left orbital gyrus, and left cerebellum anterior lobe (Fig. 1B and Table 2).

Compared to the HC group, MDD patients showed increased fALFF in bilateral post cingulum/thalamus, right orbital gyrus, and right inferior frontal gyrus, and decreased fALFF in the left fusiform gyrus and right middle frontal gyrus (Fig. 1C and Table 2). After controlling for CTQ as a covariate, MDD patients had lower fALFF in the left fusiform gyrus and right middle frontal gyrus (Fig. 1D and Table 2).

### Correlation analysis

**Figure 2:**
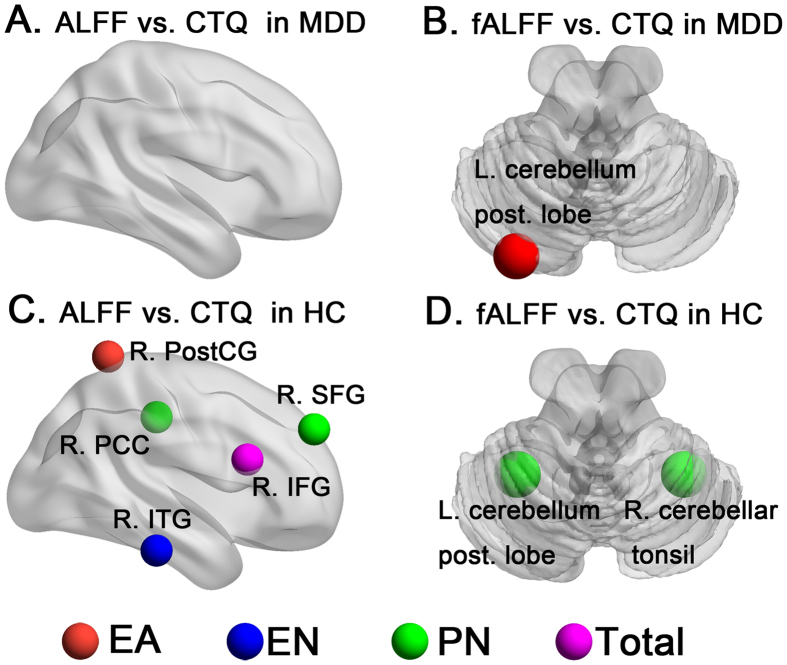
The correlation analysis of regional brain activity with CTQ in MDD and HC respectively. Figure 2A indicates correlation results of ALFF with CTQ in MDD. Figure 2B indicates correlation results of fALFF with CTQ in MDD. Figure 2C indicates correlation results of ALFF with CTQ in HC. Figure 2D indicates correlation results of fALFF with CTQ in HC (P < 0.05, AlphaSim corrected).

In the MDD group, a positive correlation was found between EA scores and brain activity in the posterior lobe of the left cerebellum (r = 0.86). There was no correlation between the other CTQ subscales or total scores and local brain activity. In the HC group, positive correlations were found between EA scores and brain activity in the right postcentral gyrus (r = 0.90); between EN scores and brain activity in the right inferior temporal gyrus (r = 0.84); between PN scores and brain activity in the right superior frontal gyrus (r = 0.79), right cingulate gyrus (r = 0.82), posterior lobe of the left cerebellum (r = 0.78), and right cerebellar tonsil (r = 0.79); and between total score and brain activity in the right inferior frontal gyrus (r = 0.86) (Table 3 and Fig. 2).

## Discussion

The results of this study demonstrate that MDD patients had higher CTQ scores (including EA, PA, EN, and PN subscales and total scores) and ALFF/fALFF in the frontal-limbic system and lower fALFF in the left fusiform gyrus and right middle frontal gyrus relative to HC. However, when CTQ scores were controlled as covariate, the bilateral amygdala, and left orbital, left fusiform, and right middle frontal gyri showed the same alterations, while other regions—including left hypothalamus, right inferior frontal gyrus, and bilateral post cingulum—did not show any more differences. In addition, the left cerebellum showed significant differences between the two groups only after controlling ELS, which also correlated with ELS in both MDD and HC. However, regions that were significantly correlated with ELS level in HC were more widely distributed than in MDD.

ELS is a significant risk factor for the development of MDD in later life^26^,^27^. Our findings indicate that MDD patients scored higher than HC on four of the five ELS subscales; scores for the sexual abuse subscale were also higher in MDD, but did not attain statistical significance. Hence, ELS is a confound in imaging data comparing differences between MDD and HC.

A meta-analysis comparing emotional or cognitive task-related and resting-state fMRI data between MDD and HC found both overlap and divergence in regions with altered brain activity associated with depression^28^. Those findings consistent with our observations were that there was greater activation in the left medial frontal lobe during the cognitive task; in the left amygdala and left parahippocampus during the emotional task; and in the left amygdala during resting state. However, alterations in trends of activation in some other regions reported by those authors did not accord with our data, such as increased and decreased activation in the right middle frontal and right inferior frontal gyrus, respectively, during the cognitive task and decreased activation in the right amygdala during the emotional task. Some of the regions identified in our study were close to those reported by others, including bilateral cingulate gyri and anterior lobe of the left cerebellum. A multivariate pattern analysis of spatial information about alterations in spontaneous brain activity in MDD reported hyperactivity/hyperconnectivity that presumably reflected the interaction of cortical midline structures with lateral prefrontal areas^29^. Indeed, we observed increased activity in the orbital and cingulate gyri, which are located exactly at the cortical midline. In addition, there have been studies comparing spontaneous neural activity measured with ALFF or fALFF between first-episode, unmedicated MDD patients and HC^23^,^30^; the variable findings from these investigations may reflect the fact that they did not take ELS into consideration, which is usually higher in MDD and may affect brain activity. Most of the regions identified in our study have been previously reported, such as the fusiform gyrus, medial frontal lobe, and cerebellum. Nonetheless, data based on larger sample sizes are needed to confirm the reliability of changes in ALFF/fALFF in MDD.

When ELS was considered as a covariate, between-groups comparisons of ALFF/fALFF changed slightly. There were fewer regions that differed between MDD and HC, suggesting that ELS is a confounding factor affecting neuroimaging findings in MDD. Regional brain activity in the right orbital and bilateral cingulate gyri may have been affected more by ELS than by MDD, as differences in these regions disappeared after regressing out ELS. Another interesting finding is that ALFF in the left cerebellum was significant only when ELS was excluded. Previous studies have indicated that even if the effects of ELS are not excluded, ALFF in cerebellum could be increased in MDD patients relative to HC^31^ and differs between treatment-resistant and -responsive depression^32^. Our data suggest that alterations in the cerebellum in MDD are neither stable nor sensitive, and may be masked by ELS. Irrespective of the impact of ELS, activity in the left orbital gyrus and bilateral amygdalae were increased and that in left fusiform and right middle frontal gyri were decreased in MDD patients, indicating that these regions are the core areas affected in this disorder.

We found that ELS levels were correlated with regional brain activity in MDD and HC, albeit in different ways. Although there are few studies that have directly measured ALFF/fALFF to assess the effects of ELS in MDD, some have reported that ALFF is altered in various emotion-related brain regions in PTSD^7^,^33^,^34^; a stronger connectivity was observed between two core DMN brain regions (posterior cingulate cortex and anterior medial prefrontal cortex) in these patients^33^. However, how different levels of ELS affect the brain remains unclear. Our data showed that the more severe the ELS, the higher the activity in the left cerebellum in MDD and in the right postcentral, inferior temporal, superior frontal, cingulate, and inferior frontal gyri and cerebellum in HC. Most of these regions are components of the prefrontal-limbic-thalamic-cerebellar circuitry, which was linked to ELS^35^.The cerebellum may be a key region that is associated with ELS in MDD as well as HC. Indeed, differences in cerebellar activity between MDD and HC appeared only after excluding ELS. One thing needs to pay attention that ELS was correlated with fALFF only in the cerebellum in MDD patients, in contrast to ALFF/fALFF correlations across many regions in HC. It is possible that ELS affects the brain in different ways in a normal or a depressed state. Early life stress could have few impact on those later develop MDD, but more impact on those develop relatively healthy in later life. In addition, although ELS is a high risk factor for later MDD, many other factors contribute to the etiology of depression, such as genetics; the heterogeneity of MDD patients may diminish the effect size of ELS. One study found no correlations between ELS and MDD in patients that had not experienced child neglect^33^, support that those MDD patients without ELS might decrease the statistic power of correlation analysis. Interestingly, we found that even within the HC group, various types of ELS differentially affected spontaneous regional brain activity. Notably, emotional stress had more obvious effects on this activity than physical stress in both MDD and HC, as measured by ALFF or fALFF. However, further study is required to determine whether the emotional component is the main factor accounting for regional alterations in brain function caused by stress. Converging evidence from animal and human studies indicated that ELS could cause persisting changes to hypothalamic–pituitary–adrenal axis (HPA) reactivity with altered cortisol responses to psychosocial stress^26^,^36^. One study pointed towards a view that the existence of reciprocal monosynaptic cerebello-hypothalamic connections and the presence of dense glucocorticoid binding sites the cerebellum plays a functional role in the regulation of HPA-axis^37^. Hence, we can speculate the physiological and pathological meaning of correlation between functional activity and ELS is that, ELS affects brain through HPA reactivity, and cerebellum plays an important role in this process. Furthermore, our data indicated most those regions correlated with ELS were contained by those with different activity between MDD and HC, which are considered important regions implicated in depressive disorder^26^, supporting a possibility that ELS heightens risk for later MDD through affecting spontaneous cerebral activity.

There were some limitations to this study. Although we tried to exclude the effects of ELS, it was only possible to do so from a methodological standpoint. Secondly, we determined the ELS level of subjects by means of a cross-sectional review in adults, and we could not exclude the possibility that known or unknown confounds had affected the brain. In addition, MDD patients may tend to recall more negative childhood experiences than HC, which would increase their ELS scores and result in a retrospective bias. More accurate information could be obtained by administering the CTQ and fMRI at different time points during an individual’s life from childhood to adulthood, in both depressed and normal states. Thirdly, the sample size was small, and therefore our conclusions require validation by additional studies with larger sample sizes.

In sum, our findings provide a neurobiological basis for how different levels of ELS correlates with brain activity in MDD and HC. There is only cerebellum showing correlation between ELS and brain activity in MDD, suggesting ELS has few impacts on MDD patients. However, when used ELS as an covariate in the group comparison, differences between MDD and HC changed partly, which might be caused by relatively diverse effects of ELS on brain in HC group. In addition, we propose that alterations in regional brain activity might be more likely to be affected by emotional than by physical ELS.

## Methods

### Subjects

First-episode, treatment-naïve MDD patients were recruited at the First Affiliated Hospital of Chongqing Medical University. HC were recruited via advertisement. A diagnosis of MDD according to DSM-IV criteria^38^ was confirmed by a structured interview conducted by two certified psychiatrists. The manual was also used to exclude other Axis I or II psychiatric disorders. Subjects were excluded from the study if they had a history of alcohol or drug abuse, neurological or serious physical diseases (e.g., gastrointestinal, neurological, endocrine, or cardiovascular disorders), morphological brain anomalies, or had any electronic or metal implants that could interfere with fMRI scanning. Written, informed consent was obtained from all subjects according to the principles of the Declaration of Helsinki (1989) and the study protocol was reviewed and approved by the Medical Ethics Committee of Chongqing Medical University. The methods were carried out in accordance with the approved guidelines.

### Assessment of ELS

ELS was quantified with the 28-item CTQ questionnaire^39^, which assesses five types of adverse childhood experience: emotional abuse (EA), physical abuse (PA), sexual abuse, emotional neglect (EN), and physical neglect (PN). Scores ranged from 5 to 25 for each subscale, with high scores indicating strong exposure to the stressor.

### fMRI data acquisition

Scans were carried out using a Signa 3.0 Tesla MRI system (GE Medical Systems, Waukesha, WI, USA) at the First Affiliated Hospital of Chongqing Medical University. Subjects were instructed to relax with their eyes closed and keep their heads still during scanning without falling asleep. At the end of the experiment, they were asked if they had fallen asleep inside the scanner during the MRI; if the answer was yes, the data were excluded. Resting-state fMRI images were collected using an EPI sequence (TR/TE = 2000/30 ms; flip angle = 90°; matrix = 64 × 64; FOV = 240 × 240 mm^2^; slice thickness/gap = 5/0 mm; and 33 axial slices to cover the whole brain), which yielded 240 brain volumes and lasted for 480 s. Three-dimensional T1-weighted anatomical images were then acquired (TR/TE = 8.35/3.27 ms; flip angle = 12°; FOV = 240 × 240 mm^2^; matrix = 256 × 256; slice thickness = 1 mm; and 156 sagittal slices).

### Image preprocessing

Data preprocessing was carried out using Data Processing Assistant for Resting-State FMRI^40^ (http://www.restfmri.net) based on Statistical Parametric Mapping (http://www.fil.ion.ucl.ac.uk/spm) and Resting-State fMRI Data Analysis Toolkit (REST)^41^ (http://www.restfmri.net). After excluding the first 10, images were corrected for slice-timing and were realigned. Data from subjects whose head motion exceeded 2 mm or for whom rotation exceeded 1° during scanning were excluded. Individual 3D T1-weighted anatomical images were co-registered to functional images. Normalized data were re-sliced at a resolution of 3 × 3 × 3 mm^3^ and spatially smoothed with a 6-mm full width at half-maximum Gaussian kernel. Functional images with linear trends were removed. Several sources of spurious variance (24 head motion parameters, averaged signals from white matter, cerebrospinal fluid, and global signals) were regressed out by multiple linear regression.

### ALFF and fALFF analysis

We computed the ALFF value of each voxel^17^ as the average square root of a given frequency (0.01–0.08 Hz) in the power spectrum. This was normalized by dividing by the global mean ALFF value. We also calculated fALFF of each voxel, defined as the ratio of the power spectrum in the low frequency (0.01–0.08 Hz) range to that of the entire frequency range^20^. The value was normalized by dividing by the global mean fALFF value.

### Statistics analysis

To evaluate ELS, a two-sample t test was used to assess differences in CTQ scores between MDD and HC. In addition, a two-sample t test (within the gray matter mask) of individual normalized ALFF/fALFF maps was used to evaluate differences in regional brain activity between MDD and HC groups, with age and sex regressed out to eliminate their respective contributions. REST software was used for the analysis. The significance threshold was set at P < 0.05 (AlphaSim corrected; combined height threshold P < 0.001 and a minimum cluster size of 17 voxels).

To control for ELS effects, a two-sample t test (within the gray matter mask) was carried out on individual normalized ALFF/fALFF maps using REST software, with age and sex regressed out to control their respective contributions. In this analysis, total CTQ scores of each subject were used as covariates. The significance threshold was set at P < 0.05 (AlphaSim corrected; combined height threshold P < 0.001 and a minimum cluster size of 17 voxels).

### Correlation analysis of regional brain activity and CTQ

To determine the relationship between regional brain activity and CTQ, we calculated Pearson’s correlation coefficients between ALFF/fALFF and CTQ scores (including total scores and five subscale scores) in a voxel-wise manner (within the gray matter mask) separately in MDD and HC groups using REST software. The statistical threshold was set at P < 0.05 (AlphaSim corrected; combined height threshold P < 0.001 and a minimum cluster size of 17 voxels).

## Additional Information

**How to cite this article**: Du, L. *et al*. Early life stress affects limited regional brain activity in depression. *Sci. Rep*. **6**, 25338; doi: 10.1038/srep25338 (2016).

## Figures and Tables

**Table 1:** Demographic and Clinical Comparisons.

	MDD (n = 16)	HC (n = 18)	T	P value
Age (years)	38.13 ± 13.19	35.33 ± 10.01	0.70	0.49
Education (years)	11.63 ± 3.612	12.83 ± 2.99	1.07	0.29
EA	10.13 ± 3.70	6.44 ± 1.75	3.63	0.002^#^
PA	7.81 ± 3.12	5.50 ± 0.71	2.90	0.010^*^
SA	6.44 ± 3.33	5.28 ± 0.70	1.37	0.19
EN	13.13 ± 4.60	9.61 ± 3.36	2.56	0.015^*^
PN	11.25 ± 4.04	7.89 ± 2.14	3.08	0.004^#^
Total Scores	48.75 ± 13.14	34.72 ± 5.05	4.02	0.001^#^

Values are mean ± s.d.

Abbreviations:

MDD: Major depressive disorder, HC: Healthy control.

EA: Emotional Abuse, PA: Physical Abuse, SA: Sexual Abuse, EN: Emotional Neglect, PN: Physical Neglect.

*P＜0.05., ^#^P＜0.01.

**Table 2:** ALFF/fALFF differences between the MDD and HC group before and after CTQ regression (AlphaSim corrected *P* < 0.05).

Brian region	BA	MNI coordinates (x y z) (mm)	Voxels	T value
*ALFF*				
Before regression				
right amygdale	34	(24, 0, −15)	148	6.20
left amygdale	28	(−21, 0, −24)	122	5.70
left orbital gyrus	11	(6, 42, −33)	147	5.94
left hypothalamus	NA	(3, −3, −12)	50	4.45
After regression				
Left Orbital Gyrus	11	(6, 39, −33)	38	6.35
left cerebellum anterior lobe	NA	(−12, −39, −21)	21	5.60
right amygdale	34	(24, 0, −15)	37	5.49
left amygdale	28	(−21, 0, −24)	20	4.82
*fALFF*				
Before regression				
right inferior frontal gyrus	38	(42, 24, −21)	20	5.53
right orbital gyrus	11	(3, 36, −33)	28	4.41
right post cingulum/thalamus	26/27	(12, −36, 9)	26	4.67
left post cingulum/thalamus	26/27	(−18, −33, 12)	21	4.99
left fusiform gyrus	37	(−42, −42, −24)	99	−6.72
right middle frontal gyrus/precentral	6	(30, −9, 51)	33	−4.89
After regression				
left inferior temporal/fusiform gyrus	37	(−42, −42, −21)	18	−4.75
right middle frontal gyrus/precentral	6	(33, −9, 45)	27	−4.74

Abbreviations: MDD: Major depressive disorder, HC: Healthy control.

x, y, z: coordinates of primary peak locations in the MNI space.

MNI: Montreal Neurological Institute.

BA: Brodmann area.

ALFF: amplitude of low-frequency fluctuation.

fALFF: fractional amplitude of low-frequency fluctuation.

**Table 3:** Association of ALFF/fALFF with CTQ scores in MDD and HC groups.

	Brian region	BA	MNI coordinates (x y z) (mm)	Voxels	r value	Alphasim P	Direction of correlation
*MDD*							
*fALFF*							
EA	left cerebellum posterior Lobe	N/A	(−27, −81, −30)	20	0.86	4.47e-005	+
*HC*							
*ALFF*							
EA	right postcentral gyrus	5	(15, −51, 72)	51	0.90	3.13e-007	+
EN	right inferior temporal gyrus	20	(60, −27, −24)	18	0.84	1.62e-005	+
PN	right superior frontal gyrus	9	(27, 51, 36)	17	0.79	9.60e-005	+
right cingulate gyrus	31	(12, −27, 42)	17	0.82	3.10e-005	+	
Total scores	right inferior frontal gyrus	48	(48, 18, 21)	22	0.86	4.49e-006	+
*fALFF*							
PN	Left cerebellum posterior Lobe	NA	(−27, −51, −54)	22	0.78	0.0001	+
right cerebellar tonsil	NA	(30, −51, −48)	20	0.79	8.35e-005	+	

MDD: Major depressive disorder, HC: Healthy control.

EA: Emotional Abuse, EN: Emotional Neglect, PN: Physical Neglect.

x, y, z: coordinates of primary peak locations in the MNI space.

MNI: Montreal Neurological Institute.

BA: Brodmann area.

ALFF: amplitude of low-frequency fluctuation.

fALFF: fractional amplitude of low-frequency fluctuation.
